# Supplementary material for: Mendelian randomization evaluation of causal effects of fibrinogen on incident coronary heart disease
Source: PLoS One. 2019 May 10;14(5):e0216222. doi: 10.1371/journal.pone.0216222 (PMC6510421; doi:10.1371/journal.pone.0216222)
Supplement: S1 File — File containing the Supplemental Online Methods (including cohort specific information) as well as the Supplemental Tables (A-E). Table A. Association between allele score (AS) and fibrinogen as well as rs1800790 (FGB -455G>A) and fibrinogen. Table contains association between AS and fibrinogen as well as the association between FGB variant rs1800790 with fibrinogen. Also given is the association between the AS and fibrinogen after rescaling (RS) the AS in each cohort so that a unit of 1 represented 50% of the range. This transformation allows the allele score associations to be more directly compared with those for rs1800790. Rs1800790 was unavailable in SHIP. The meta-analysis represents a fixed effects meta-analysis Table B. Comparison of allele score and rs1800790 as genetic instruments for CHD. Comparison of the allele score vs rs1800790 as a genetic instrument for CHD. 2SC = two-stage Cox (2SC) model; CI = 95% confidence interval; HR = Hazard Ratio; MR = Mendelian Randomization; OR = Odds Ratio; Q = Cochran’s Q; SE = standard error. Table C. Estimates of causal effect of fibrinogen on metabolic CHD risk factors. As body mass index was the coronary heart disease (CHD) trait with the most evidence for pleiotropic effects from our allele score we used 3 Mendelian Randomization (MR) methods from as implemented in MR-base (www.mrbase.org)[4]. MR-base uses published genome-wide association studies (GWAS) to perform 2-sample MR. For the genetic variant-fibrinogen associations we used the most recently published fibrinogen GWAS.[5] References and sample sizes for each of the CHD risk factor outcomes appear in the table. Only variants that were a part of the allele score were used in the MR analyses, with each GWAS having between 23 and 32 of the 38 variants represented. HOMA-IR = homeostatic model assessment insulin resistance; LDL = low-density lipoprotein, OR = odds ratio; SD = standard deviation; SE = standard error; SNP = single nucleotide polymorphism. Table D. A [file pone.0216222.s001.doc]

**Supplemental Tables**

**Table A. Association between allele score (AS) and fibrinogen as well as rs1800790 (FGB -455G>A) and fibrinogen**.

|  | AS-Fibrinogen Association | AS-Fibrinogen (RS) Association | AS-Fibrinogen Association P | AS-Fibrinogen (RS) P | rs1800790–Fibrinogen Association | rs1800790–Fibrinogen P |
| --- | --- | --- | --- | --- | --- | --- |
| KORA | 0.01 | 0.17 | 2.53E-06 | 2.53E-06 | 0.10 | 5.27E-09 |
| GENOA | 0.03 | 0.47 | 1.05E-04 | 1.05E-04 | 0.00 | 9.55E-01 |
| ARIC | 0.01 | 0.20 | 1.52E-15 | 1.52E-15 | 0.07 | 5.17E-11 |
| CHS | 0.02 | 0.25 | 1.25E-11 | 1.25E-11 | 0.10 | 5.87E-08 |
| GeneSTAR | 0.04 | 0.57 | 3.11E-05 | 3.11E-05 | 0.11 | 1.58E-01 |
| FHS | 0.03 | 0.35 | 8.78E-13 | 8.78E-13 | 0.12 | 5.91E-06 |
| RS | 0.03 | 0.49 | 1.13E-06 | 1.13E-06 | 0.19 | 5.27E-04 |
| MESA | 0.02 | 0.26 | 4.61E-08 | 4.61E-08 | 0.11 | 9.88E-06 |
| LURIC | 0.02 | 0.24 | 8.62E-03 | 8.62E-03 | -0.07 | 1.67E-01 |
| SHIP | 0.01 | 0.16 | 1.66E-04 | 1.66E-04 |  |  |
| Meta-Analysis | 0.02 | 0.28 | 1.51E-123 | 2.46E-122 | 0.10 | 1.12E-70 |

Table contains association between AS and fibrinogen as well as the association between *FGB* variant rs1800790 with fibrinogen. Also given is the association between the AS and fibrinogen after rescaling (RS) the AS in each cohort so that a unit of 1 represented 50% of the range. This transformation allows the allele score associations to be more directly compared with those for rs1800790. Rs1800790 was unavailable in SHIP. The meta-analysis represents a fixed effects meta-analysis

**Table B. Comparison of allele score and rs1800790 as genetic instruments for CHD**

| **Model** | **Instrument** | **Beta** | **SE** | **HR** | **CI** | **P** | **Q** | **P(Q)** |
| --- | --- | --- | --- | --- | --- | --- | --- | --- |
| 2SC | allele score | 0.52 | 0.19 | 1.66 | 1.06, 2.58 | 0.006 | 4.91 | 0.84 |
| 2SC | rs1800790 | -0.03 | 0.27 | 0.97 | 0.58, 1.63 | 0.90 | 5.93 | 0.66 |

Comparison of the allele score vs rs1800790 as a genetic instrument for CHD. 2SC = two-stage Cox (2SC) model; CI = 95% confidence interval; HR = Hazard Ratio; MR = Mendelian Randomization; OR = Odds Ratio; Q = Cochran’s Q; SE = standard error

**Table C. Estimates of causal effect of fibrinogen on metabolic CHD risk factors**

| Risk Factor | Risk Factor units | MR Method | N SNPs | Beta | SE | P | Risk Factor N |
| --- | --- | --- | --- | --- | --- | --- | --- |
| Body mass index[6] | SD (kg/m^2^) | MR Egger | 27 | -0.13 | 0.28 | 0.64 | 339,224 |
| Body mass index[6] | SD (kg/m^2^) | Weighted median | 27 | -0.05 | 0.13 | 0.71 | 339,224 |
| Body mass index[6] | SD (kg/m^2^) | Inverse variance weighted | 27 | -0.03 | 0.12 | 0.83 | 339,224 |
| Waist circumference[7] | SD (cm) | MR Egger | 27 | -0.01 | 0.23 | 0.95 | 224,459 |
| Waist circumference[7] | SD (cm) | Weighted median | 27 | -0.02 | 0.14 | 0.88 | 224,459 |
| Waist circumference[7] | SD (cm) | Inverse variance weighted | 27 | -0.13 | 0.10 | 0.23 | 224,459 |
| Waist-to-hip ratio[7] | SD | MR Egger | 27 | 0.06 | 0.23 | 0.81 | 224,459 |
| Waist-to-hip ratio[7] | SD | Weighted median | 27 | 0.02 | 0.15 | 0.90 | 224,459 |
| Waist-to-hip ratio[7] | SD | Inverse variance weighted | 27 | -0.05 | 0.10 | 0.61 | 224,459 |
| LDL cholesterol[8] | SD (mg/dL) | MR Egger | 23 | 0.17 | 0.69 | 0.81 | 173,082 |
| LDL cholesterol[8] | SD (mg/dL) | Weighted median | 23 | 0.16 | 0.19 | 0.40 | 173,082 |
| LDL cholesterol[8] | SD (mg/dL) | Inverse variance weighted | 23 | 0.51 | 0.31 | 0.10 | 173,082 |
| Triglycerides[8] | SD (mg/dL) | MR Egger | 23 | -0.26 | 0.45 | 0.57 | 177,861 |
| Triglycerides[8] | SD (mg/dL) | Weighted median | 23 | -0.21 | 0.18 | 0.23 | 177,861 |
| Triglycerides[8] | SD (mg/dL) | Inverse variance weighted | 23 | -0.10 | 0.20 | 0.62 | 177,861 |
| HOMA-IR[9] | log(HOMA) | MR Egger | 26 | 0.00 | 0.23 | 0.99 | 46,186 |
| HOMA-IR[9] | log(HOMA) | Weighted median | 26 | 0.00 | 0.14 | 1.00 | 46,186 |
| HOMA-IR[9] | log(HOMA) | Inverse variance weighted | 26 | -0.17 | 0.11 | 0.13 | 46,186 |
| Type 2 diabetes[10] | log(OR) | MR Egger | 32 | -1.16 | 2.01 | 0.57 | 120,286 |
| Type 2 diabetes[10] | log(OR) | Weighted median | 32 | 0.00 | 1.31 | 1.00 | 120,286 |
| Type 2 diabetes[10] | log(OR) | Inverse variance weighted | 32 | 0.43 | 0.85 | 0.62 | 120,286 |

As body mass index was the coronary heart disease (CHD) trait with the most evidence for pleiotropic effects from our allele score we used 3 Mendelian Randomization (MR) methods from as implemented in MR-base ([www.mrbase.org](http://www.mrbase.org/))[4]. MR-base uses published genome-wide association studies (GWAS) to perform 2-sample MR. For the genetic variant-fibrinogen associations we used the most recently published fibrinogen GWAS.[5] References and sample sizes for each of the CHD risk factor outcomes appear in the table. Only variants that were a part of the allele score were used in the MR analyses, with each GWAS having between 23 and 32 of the 38 variants represented. HOMA-IR = homeostatic model assessment insulin resistance; LDL= low-density lipoprotein, OR = odds ratio; SD = standard deviation; SE = standard error; SNP = single nucleotide polymorphism

**Table D. Allele Score Variants**

|  | Locus | Closest Gene | MESA | WGHS | LURIC | SHIP | Study | Published Direction of Association |
| --- | --- | --- | --- | --- | --- | --- | --- | --- |
| rs1892534 | 1p31.3 | *LEPR* |  |  |  |  | de Vries et al | -1 |
| rs10157379 | 1q44 | *NLRP3* |  |  |  |  | de Vries et al | -1 |
| rs7588285 | 2p25.3 | *COLEC11* |  |  |  |  | deVries et al | 1 |
| rs1558643 | 2q12 | *IL1R1* |  |  |  |  | deVries et al | 1 |
| rs6734238 | 2q13 | *IL1F10* |  |  |  |  | deVries et al | 1 |
| rs1476698 | 2q27.3 | *FARP2* |  |  |  |  | Sabater-Lleal et al | 1 |
| rs715 | 2q34 | *CPS1* |  |  |  |  | de Vries et al | -1 |
| rs59104589 | 2q37.3 | *HDLBP* |  |  |  |  | de Vries et al | -1 |
| 3:129228166 | 3p21.3 | *IFT122* |  |  | NA | NA | de Vries et al | 1 |
| rs62246343 | 3p25.3 | *LHFPL4* |  |  |  |  | de Vries et al | 1 |
| rs1976714 | 3q21.1 | *PDIA5* |  |  |  |  | de Vries et al | -1 |
| rs16844401 | 4p16.3 | *HGFAC* |  |  |  |  | Sabater-Lleal et al | 1 |
| rs59950280 | 4p16.3 | *HGFAC* |  |  |  |  | de Vries et al | 1 |
| rs148685782 | 4q31.3 | *FGG* | NA | NA | NA | NA | Huffman et al | -1 |
| rs7439150 | 4q31.3 | *FGB* |  |  |  |  | de Vries et al | 1 |
| 7:150289652 | 7q36.1 | *GIMAP4* |  |  |  | NA | de Vries et al | -1 |
| rs11780978 | 8q24.3 | *PLEC* |  |  |  |  | de Vries et al | 1 |
| rs3138493 | 9q22.2 | *GADD45G* |  |  |  |  | de Vries et al | -1 |
| rs7916868 | 10q21.3 | *JMJD1C* |  |  |  |  | de Vries et al | 1 |
| rs7934094 | 11p12 | *TTC17* |  |  |  |  | de Vries et al | -1 |
| rs11230201 | 11q12.2 | *MS4A6A* |  |  |  |  | de Vries et al | -1 |
| rs7232 | 11q12.2 | *MS4A6A* |  |  |  | NA | de Vries et al | -1 |
| 12:21703935 | 12p12.1 | *GSY2* |  |  |  | NA | de Vries et al | 1 |
| rs2731439 | 12q13.12 | *DIP2B* |  |  |  |  | de Vries et al | -1 |
| rs367677 | 14q24.1 | *ZFP36L1* |  |  |  |  | de Vries et al | 1 |
| rs434943 | 14q24.1 | *ACTN1* |  |  |  |  | Sabater-Lleal et al | 1 |
| rs56702977 | 15q15.1 | *CAPN3* |  |  |  |  | de Vries et al | 1 |
| rs12913259 | 15q21.2 | *SPPL2A* |  |  |  |  | de Vries et al | -1 |
| rs12915708 | 15q21.2 | *SPPL2A* |  |  |  |  | Sabater-Lleal et al | -1 |
| rs11859517 | 16q12.2 | *CHD9* |  |  |  |  | de Vries et al | -1 |
| rs1035560 | 16q22.2 | *PKD1L3* |  |  |  |  | de Vries et al | 1 |
| rs7224737 | 17q21.2 | *RAB5C* |  |  |  |  | de Vries et al | 1 |
| rs10512597 | 17q25.1 | *CD300LF* |  |  |  |  | Sabater-Lleal et al | -1 |
| rs1800961 | 20q13.12 | *HNF4A* |  |  |  |  | de Vries et al | -1 |
| rs4817986 | 21q22.2 | *PSMG1* |  |  |  |  | Sabater-Lleal et al | -1 |
| rs9808651 | 21q22.2 | *PSMG1* |  |  |  |  | de Vries et al | -1 |
| rs6010044 | 22q13.33 | *SHANK3* |  |  |  |  | Sabater-Lleal et al | -1 |
| rs75347843 | 22q13.33 | *SHANK3* | NA | NA | NA | NA | de Vries et al | 1 |

Variants which composed the allele score along with their availability in each cohort as well as selection from either the 1000 Genomes imputation GWAS by de Vries et al,[1] the meta-analysis by Sabater-Lleal et al,[2] or the rare and low-frequency variant meta-analysis by Huffman et al,[3] and the published direction of association. NA indicates that the variant was not available. All variants were available for all discovery cohorts by design, thus only the replication cohorts (MESA, WGHS, LURIC, and SHIP) are listed where one or more variants may have been missing. Closest Gene = gene annotation based on location within a gene or the closest gene for intergenic variants; Locus = genomic location; Published Direction of Association = direction of association for the variant in the given study; Study = published study variant was taken from

**Table E. Association of allele score with CHD risk factors**

|  | Beta | SE | P | Q | P(Q) |
| --- | --- | --- | --- | --- | --- |
| Smoking | 0.005 | 0.002 | 0.02 | 6.15 | 0.80 |
| BMI | 0.015 | 0.006 | 0.01 | 10.2 | 0.43 |
| Hypertension | 0.001 | 0.002 | 0.65 | 9.49 | 0.49 |
| HDL | -0.003 | 0.003 | 0.36 | 22.2 | 0.01 |
| LDL | 0.013 | 0.007 | 0.07 | 15.7 | 0.11 |
| Type II Diabetes | -0.003 | 0.005 | 0.49 | 6.13 | 0.80 |

Fixed effects meta-analysis for association between fibrinogen and CHD risk factors. Fixed effects meta-analysis was used for all associations despite some evidence for heterogeneity for HDL P(Q) < 0.05. The association was still not significant in the random effects meta-analysis. Hypertension and Type II Diabetes were binary variables. Smoking was a categorical outcome for current, former, or never smokers. Beta = meta-analysis effect estimate; SE = meta-analysis standard error; P = meta-analysis P; Q = Cochran's Q; P(Q) = P-value associated with Cochran's Q

**Supplemental Online Methods**

**Cohort descriptions in alphabetical order**

**Atherosclerosis Risk in Communities Study (ARIC)**

*Study participants*

The Atherosclerosis Risk in Communities (ARIC) study has been described in detail previously.[11] Briefly, participants aged 45 to 64 years at baseline were recruited from four communities: Forsyth County, North Carolina; Jackson, Mississippi; Minneapolis, Minnesota; and Washington County, Maryland. A total of 15,792 individuals, predominantly of European and African ancestry, participated in the baseline examination in 1987-1989, with three additional triennial follow-up examinations, a fifth exam in 2011-2013, and a sixth exam in 2016-2017.

*MI and CHD Assessment*

Non-fatal and fatal myocardial infarction (MI) and coronary heart disease (CHD) events are monitored by community surveillance of death certificates and hospital discharge records. Participants are also contacted annually to obtain information on hospitalizations. Interviews with next of kin and questionnaires completed by physicians and medical examiners or coroners were used to collect information on deaths, and review and abstraction of hospital records were used to collect information on possible fatal and nonfatal events. Events were classified using standardized and published criteria.[12]

*Fibrinogen Measurement*

Fibrinogen was assayed in participants after an eight hour fasting period.[13, 14] Fibrinogen was measured with the thrombin-time titration method[15] with reagents and calibration materials (Fibriquik) obtained from General Diagnostics (Organon-Technika Co). Fibrinogen is reported in g/L.

*Genotyping and imputation*

Samples were genotyped on Affymetrix Genome-Wide Human SNP Array 6.0. Imputation was performed in two steps: (1) Pre-phasing with ShapeIt (v1.r532 ) (2) Imputation with IMPUTE2. Measured SNPs used for imputation were restricted to have: MAF >0.005, >95% complete, HWE > 0·00001. Final imputations were carried out using IMPUTE2 using the reference panel: 1,000 Genomes haplotypes -- Phase I integrated variant set release (v3) in NCBI build 37 (hg19) in chunks of size 5 Mb. All 1092 individuals were used for the imputation from the reference panel. Variants from the imputation were reported in variant dosage format.

*Acknowledgments*

The Atherosclerosis Risk in Communities study has been funded in whole or in part with Federal funds from the National Heart, Lung, and Blood Institute, National Institutes of Health, Department of Health and Human Services (contract numbers HHSN268201700001I, HHSN268201700002I, HHSN268201700003I, HHSN268201700004I and HHSN268201700005I), R01HL087641, R01HL59367 and R01HL086694; National Human Genome Research Institute contract U01HG004402; and National Institutes of Health contract HHSN268200625226C. The authors thank the staff and participants of the ARIC study for their important contributions. Infrastructure was partly supported by Grant Number UL1RR025005, a component of the National Institutes of Health and NIH Roadmap for Medical Research.

**Cardiovascular Health Study (CHS)**

*Study participants*

The Cardiovascular Health Study (CHS) is a population-based cohort study of cardiovascular risk factors, including myocardial infarction (MI) and coronary heart disease (CHD), in adults ≥65 years conducted across four field centers.[16] The original predominantly Caucasian cohort of 5,201 persons was recruited in 1989-1990 from random samples of the Medicare eligibility lists; subsequently, an additional predominantly African-American cohort of 687 persons were enrolled for a total sample of 5,888.

*MI and CHD Assessment*

CHS participants were seen in clinic annually from 1989 through 2009, and were contacted by phone at 6-month intervals between clinic visits and after clinic visits ceased. Information was collected about hospitalizations and potential cardiovascular events during these clinic and phone visits. The reported events were further investigated, with detailed data abstracted for adjudication of events by events committees. Myocardial infarction (MI) was adjudicated to have occurred in the presence of evolving Q-wave MI or cardiac pain plus abnormal enzymes and either an evolving ST-T pattern or new left bundle branch block.[16] CHD was not directly adjudicated, but is a composite variable defined for the current analysis as the occurrence of an MI, angioplasty, coronary artery bypass graft, or CHD death.[17]

*Fibrinogen Measurement*

Plasma fibrinogen was measured using a BBL fibrometer (Becton Dickinson, Cockeysville, MD) by the Clauss method[15] with Dade fibrinogen calibration reference (Baxter-Dade, Bedford, MA) and bovine thrombin (Parke-Davis, Lititz, PA).[18] Fibrinogen is reported in g/L.

*Genotyping and Imputation*

DNA was extracted from blood samples drawn on all participants at their baseline examination, and was genotyped on CHS participants who were free of cardiovascular disease at baseline, consented to genetic testing, and had DNA available for genotying. Genotyping was performed at the General Clinical Research Center's Phenotyping/Genotyping Laboratory at Cedars-Sinai using the Illumina 370CNV BeadChip system. Variants with a call rate less than or equal to 0.95 were excluded.

Prior to imputation, cleaned genotypes from  the Illumina CNV370 were merged with genotypes from ITMAT-Broad-CARe (IBC) Illumina iSELECT chip. Imputation was performed using the 1000 Genomes Project phase 1 version 3 reference using minimac (release stamp 2012-11-16). Variants from the imputation were reported in variant dosage format. SNPs were excluded for variance on the allele dosage <=0.01.

*Consent*

CHS was approved by institutional review committees at each site, the subjects gave informed consent, and those included in the present analysis consented to the use of their genetic information for the study of cardiovascular disease.

*Acknowledgments*

This CHS research was supported by NHLBI contracts HHSN268201200036C, HHSN268200800007C, N01HC55222, N01HC85079, N01HC85080, N01HC85081, N01HC85082, N01HC85083, N01HC85086; and NHLBI grants U01HL080295, R01HL087652, R01HL105756, R01HL103612, R01HL120393, and R01HL130114 with additional contribution from the National Institute of Neurological Disorders and Stroke (NINDS). Additional support was provided through R01AG023629 from the National Institute on Aging (NIA). A full list of principal CHS investigators and institutions can be found at CHS-NHLBI.org.

The provision of genotyping data was supported in part by the National Center for Advancing Translational Sciences, CTSI grant UL1TR000124, and the National Institute of Diabetes and Digestive and Kidney Disease Diabetes Research Center (DRC) grant DK063491 to the Southern California Diabetes Endocrinology Research Center.

The content is solely the responsibility of the authors and does not necessarily represent the official views of the National Institutes of Health.

**Framingham Heart Study (FHS)**

*Study Participants*

The FHS is a three generational prospective cohort that has been described in detail previously.[19, 20] Individuals were initially recruited in 1948 in Framingham, USA to evaluate cardiovascular disease risk factors. The second generation cohort (5,124 offspring of the original cohort) was recruited between 1971 and 1975. The third generation cohort (4,095 grandchildren of the original cohort) was collected between 2002 and 2005. As FHS contains families only unrelated probands were used for this analysis while focusing on maximizing the number of cases.

*MI and CHD assessment*

In the Framingham Heart Study, coronary heart disease (CHD) was defined as a fatal coronary event or myocardial infarction (MI). All study participants were under continuous surveillance for the development of CHD events and death, and information about CHD events on follow‐up was obtained with the aid of medical histories, physical examinations at the study clinic, hospitalization records, and communication with personal physicians. All suspected new events were reviewed by a panel of 3 experienced investigators who evaluated all pertinent medical records. A separate review committee that included a neurologist adjudicated cerebrovascular events, and a heart study neurologist examined most participants with suspected stroke. CHD and MI incident status was determined from the exam where fibrinogen was measured until the most recent examination cycle for Offspring (exam 9: 2011-2014) and third generation (exam 2: 2008-2011) cohorts.

*Fibrinogen measurement*

Fibrinogen was measured at exam 6 of the Offspring (1995-1998) and exam 1 of the third generation (2002-2005) cohorts, using the Clauss method.[15]

*Genotyping and Imputation*

Genotyping was carried out as a part of the SNP Health Association Resource project using the Affymetrix 500K mapping array (250K Nsp and 250K Sty arrays) and the Affymetrix 50K supplemental gene focused array on 9274 individuals. 412 053 SNPs passed all quality control parameters (SNP call rate >95%, MAF>0.01 and HWE P >1E-6, individual call rate > 97%) to be used for imputation. Imputation of ~35 million SNPs using the 1000 Genomes Project phase I version 3 (ALL) reference panel was conducted using the algorithm implemented in minimach. SNPs used for calculating the genetic risk score were taken from this 1000 Genomes imputed dataset or directly genotyped using the Illumina HumanExome BeadChip array.

*Acknowledgements*

The NHLBI’s Framingham Heart Study is a joint project of the National Institutes of Health and Boston University School of Medicine and was supported by contract no. N01-HC-25195 and contract no. N02-HL-6-4278 (contract with Affymetrix, Inc for genotyping services). Genotyping, quality control and calling of the Illumina HumanExome BeadChip in the Framingham Heart Study was supported by funding from the National Heart, Lung and Blood Institute Division of Intramural Research (Daniel Levy and Christopher J. O’Donnell, Principle Investigators). Support for the centralized genotype calling was provided by Building on GWAS for NHLBI-diseases: the U.S. CHARGE consortium through the National Institutes of Health (NIH) American Recovery and Reinvestment Act of 2009 (5RC2HL102419). A portion of this research was conducted using the Linux Clusters for Genetic Analysis (LinGA) computing resources at Boston University Medical Campus.

**Genetic Epidemiology Network of Arteriopathy (GENOA):**

*Study participants*

GENOA is one of four networks in the NHLBI Family-Blood Pressure Program (FBPP).[21] GENOA's long-term objective is to elucidate the genetics of target organ complications of hypertension, including both atherosclerotic and arteriolosclerotic complications involving the heart, brain, kidneys, and peripheral arteries. The longitudinal GENOA Study recruited European-American and African-American sibships with at least 2 individuals with clinically diagnosed essential hypertension before age 60 years. All other members of the sibship were invited to participate regardless of their hypertension status. Participants were diagnosed with hypertension if they had either 1) a previous clinical diagnosis of hypertension by a physician with current anti-hypertensive treatment, or 2) an average systolic blood pressure ≥ 140 mm Hg or diastolic blood pressure ≥ 90 mm Hg based on the second and third readings at the time of their clinic visit. Exclusion criteria were secondary hypertension, alcoholism or drug abuse, pregnancy, insulin-dependent diabetes mellitus, or active malignancy. During the first exam (1995-2000), 1,583 European Americans from Rochester, MN and 1,854 African Americans from Jackson, MS were examined. Between 2000 and 2005, 1,241 of the European Americans and 1,482 of the African Americans returned for a second examination.

The current study was limited to European Americans recruited at the Mayo Clinic in Rochester, MN. Only unrelated probands were used for the analysis focusing on maximizing the number of cases. Fibrinogen levels, incident event data, and GWAS data were available for 417 participants.

*MI and CHD Assessment*

We used the electronic medical record (EMR) at the Mayo Clinic to determine incident cases of CHD and MI among GENOA participants who had no history of CHD or MI at the time of their second GENOA examination. CHD cases were identified as those assigned an ICD-9 code of 410-414 on at least two separate clinical visits after their second GENOA examination. MI cases were identified as those assigned an ICD-9 code of 410 on at least two separate clinical visits after their second GENOA examination. We excluded all participants who had at least one ICD-9 of 410-414 in their EMR before their second GENOA examination. We used the date of their first relevant ICD-9 code to calculate a participant’s time to event. For those without at least two of these ICD-9 codes, we used the date of the last information for them in the EMR as their date for censoring.

*Fibrinogen Measurement*

Fibrinogen levels as well as all risk factors considered in the current study were measured at the second GENOA examination. Blood was drawn after an overnight fast. Fibrinogen was measured by the Clauss (clotting time-based) method.[15] Fibrinogen is reported in g/L.

*Genotyping and imputation*

1,386 participants of European ancestry were genotyped using the Affymetrix® Genome-Wide Human SNP Array 6.0 platform and 126 participants were genotyped using the Illumina® Human1M-Duo Bead Chip. Participants were excluded if they had an overall SNP call rate <95%, sex mismatch between genotypic and phenotypic measurement, or low identity-by-state with all other participants. SNPs were excluded if they had unknown chromosomal location, a call rate less than 95% or a minor allele frequency (MAF) less than 0.01.

Genotypes were imputed to the 1000 Genomes Phase I integrated variant set (NCBI build 37 / hg19) using the IMPUTE2 software. Since GENOA is composed of sibships, we calculated PCs to control for population stratification in an unrelated sample of participants. First, we removed SNPS that had poor imputation quality as measured by the estimated r^2^ between imputed and true genotypes (r^2^<0.8). Next, we obtained the maximum number of unrelated individuals in our total sample by selecting one sibling randomly from each sibship (N= 570). In this sub-sample, we calculated the first ten PCs on the set of SNPs that were common to both genotyping platforms (Affymetrix 6.0 and Illumina 1M-Duo) and were also in HapMap in order to ensure no missing values for SNPs. We then used the loading matrix for these PCs to calculate the PC values in the full sample. Next, outliers of more than 6 standard deviations on any of the ten PCs were removed to ensure that the PCs were not capturing variation due to poor quality genotyping or single individuals with a dramatically different admixture profile than the remainder of the sample. A total of 45 participants were removed from the full sample. Next, we again selected an unrelated sub-sample of participants by randomly selecting one participant from each sibship (N=556) and recalculated the first ten PCs in this sample. Finally, we used the loading matrix to calculate the first ten PCs in the final sample.

*Consent*

Study protocols were approved by the University of Michigan and Mayo Clinic Institutional Review Boards and participants gave written informed consent. Participants included in the present analysis consented to the use of their genetic information for the study of cardiovascular disease. Participants also consented for use of their clinical record in research studies.

*Acknowledgments*

Support for GENOA was provided by the National Heart, Lung and Blood Institute (HL054464; HL054481; HL087660; HL119443) of the National Institutes of Health. Genotyping was performed at the Mayo Clinic (Stephen T. Turner, MD, Mariza de Andrade PhD, Julie Cunningham, PhD). We thank Eric Boerwinkle, PhD and Megan L. Grove, MS from the Human Genetics Center and Institute of Molecular Medicine and Division of Epidemiology, University of Texas Health Science Center, Houston, Texas, USA for their help with genotyping. We would also like to thank the families that participated in the GENOA study.

**Genetic Study of Atherosclerosis Risk (GeneSTAR)**

*Study participants*

The Genetic Study of Atherosclerosis Risk (GeneSTAR) is a European and African American family-based cohort study, identified from probands with documented premature (<age 60) coronary heart disease (CHD) events in one of 10 Baltimore, Maryland, USA hospitals between 1982 and 2006. Their apparently healthy siblings < 60 years of age and free of known CHD were recruited and screened from 1983 to 2007, and apparently healthy offspring of the probands or siblings and the coparents of the offspring were recruited and screened from 2003-2007. Baseline screening included questionnaires on medical history, medication use, smoking and other lifestyle factors, physical examination and measurements, and the collection of fasting blood samples.[22] For this analysis, only unrelated European Americans were included (N = 594).

*MI and CHD Assessment*

Participants completed a standardized health status and cardiovascular disease event questionnaire at approximately five-year intervals after their baseline visit with trained telephone interviewers between 1992 and 2012. For deceased siblings, proxy interviews were completed with the next of kin, and death certificates were obtained. Medical records were then obtained for all reports of a CHD event, any possibly related diagnosis, diagnostic procedure (including exercise tests, thallium imaging, or coronary angiography) or therapeutic procedure, including percutaneous coronary intervention (PCI) or coronary artery bypass graft (CABG). Incident CHD was defined as sudden cardiac death, definite or probable MI, or coronary revascularization procedures (CABG or PCI).

*Fibrinogen Measurement*

Plasma fibrinogen was measured using a modified Clauss method[15] on an automated optical clot detection device (Dade-Behring, Newark, DE). Excess thrombin was added to citrated plasma, and the time needed for clot formation was recorded. This clotting time was then compared with that of a standardized fibrinogen preparation. Fibrinogen is reported here as g/L.

*Genotyping and Imputation*

Samples were genotyped with the Illumina Human 1M_v1C array at deCODE Genetics in Reykjavik, Iceland and with the Illumina HumanExome BeadChip v1.2 through the RS&G Service at the Northwest Genomics Center at the University of Washington, Seattle. Variants with call rate < 90% were excluded. Imputation was performed using the 1000 Genomes Project phase I version 3 cosmopolitan reference panel (March 2012) using IMPUTE2. Variants from the imputation were reported in variant dosage format.

*Acknowledgements*

GeneSTAR was supported by grants from the National Institutes of Health/National Heart, Lung, and Blood Institute (U01 HL72518, HL087698, HL49762, HL59684, HL58625, HL071025, HL112064), by a grant from the National Institutes of Health/National Institute of Nursing Research (NR0224103), and by a grant from the National Institutes of Health/National Center for Research Resources (M01-RR000052) to the Johns Hopkins General Clinical Research Center. Genotyping services for the Exome Chip were provided through the RS&G Service by the Northwest Genomics Center at the University of Washington, Department of Genome Sciences, under U.S. Federal Government contract number HHSN268201100037C from the National Heart, Lung, and Blood Institute.

**Cooperative Health Research in the Region of Augsburg (KORA)**

*Study participants*

The Cooperative Health Research in the Region of Augsburg, Germany Survey 4 (KORA S4) is a population based survey of 4,261 individuals recruited from Augsburg, Germany from October, 1999 – April, 2001. All participants completed a detailed questionnaire which gathered information on medical history, clinical, and lifestyle factors. Peripheral blood samples were taken for later analyses.[23] The collection and analysis of all KORA data was approved by the ethics committee of the Bavarian Medical Association in Munich, Germany.

*MI and CHD Assessment*

Non-fatal MI events were reported to the Coronary Events Registry and linked to the cohorts using name and date of birth.[24, 25] For individuals who moved out of the study area, mailed questionnaires and general practitioner notes were used to validate MI. In the event that the questionnaires were not returned the date of move was used as the loss to follow-up date. Fatal MI events found in population registries but not the Coronary Events Registry were classified using the general practitioner’s notes, hospital discharge letter, or ICD-9 code of the underlying cause of death. CHD was only available by self-report taken in response to the questionnaire administered during enrollment into the KORA S4 survey, and thus was not used for this analysis.

*Fibrinogen Measurement*

Fibrinogen was assayed via an immunonephelometric method (Dade Behring Marburg GmbH, Marburg, Germany) with a Behring Nephelometer II analyzer. Fibrinogen is reported in g/L.

*Genotyping and imputation*

Samples were genotyped on an Affymetrix Axiom array. Variants with a call rate less than 0.98, Hardy-Weinberg Equilibrium P < 5x10^-6^, and MAF < 0.01 were removed. Imputation was performed using the 1000 Genomes Project phase I version 3 reference panel with IMPUTE 2.3.0. Phasing of the data was performed using SHAPEIT v2. Variants from the imputation were reported in variant dosage format.

*Acknowledgments*

We would like to acknowledge all KORA participants and staff who contributed to this project. The KORA study was initiated and financed by the Helmholtz Zentrum München – German Research Center for Environmental Health, which is funded by the German Federal Ministry of Education and Research (BMBF) and by the State of Bavaria. Furthermore, KORA research was supported within the Munich Center of Health Sciences (MC-Health), Ludwig-Maximilians-Universität, as part of LMUinnovativ. Part of this work was financed by the German National Genome Research Network (NGFNPlus, project number 01GS0834) and through additional funds from the University of Ulm. This work was supported in part by the German Federal Ministry of Education and Research (BMBF) within the framework of the e:Med research and funding concept (e:AtheroSysMed).

**Ludwigshafen Risk and Cardiovascular Health (LURIC)**

*Study participants*

The Ludwigshafen Risk and Cardiovascular Health (LURIC) study is a monocentric hospital based prospective study including 3316 individuals referred for coronary angiography recruited in the Ludwigshafen Cardiac Center, southwestern Germany from 1997 – 2000.[26] Clinical indications for angiography were chest pain or a positive non-invasive stress test suggestive of myocardial ischemia (MI). To limit clinical heterogeneity, individuals suffering from acute illnesses other than acute coronary syndrome, chronic non-cardiac diseases and a history of malignancy within the five past years were excluded. All participants were completed a detailed questionnaire which gathered information on medical history, clinical, and lifestyle factors. Fasting blood samples were obtained by venipuncture in the early morning and stored for later analyses.

Information on vital status during follow-up was obtained from local registries. Death certificates, medical records of local hospitals, and autopsy data were reviewed independently by two experienced clinicians who were blinded to patient characteristics and who classified the causes of death.

*MI and CHD Assessment*

Coronary heart disease (CHD) at baseline was defined as the presence of a visible luminal narrowing (>50% stenosis) in at least one of 15 coronary segments according to the classification of the American Heart Association. These patients were excluded from the analysis. Incident CHD was defined as death due to CHD, including: death due to sudden cardiac death, fatal myocardial infarction, death after intervention to treat coronary heart disease, or other death due to CHD.

*Fibrinogen Measurement*

Fibrinogen was with the Clauss method[15] (STA fibrinogen/STA Stago, Stago Diagnostica, Roche Mannheim, Germany). For a total of 921 individuals fibrinogen was measured but only for 907 individuals genotypes were also available. Median follow-up was 10.2 (9.61-10.8) years.

*Genotyping and imputation*

Samples were genotyped on an Affymetrix 6.0 array. Variants with a call rate less than 0.98, Hardy-Weinberg Equilibrium P < 5x10^-4^, and MAF < 0.01 were removed. Imputation was performed using the 1000 Genomes Project phase I version 3 reference panel with IMPUTE 2.

*Consent*

Study protocols were approved by the ethics committee of the "Landesärztekammer Rheinland-Pfalz" and the study was conducted in accordance with the "Declaration of Helsinki”. Informed written consent was obtained from all participants.

*Acknowledgement*

We thank the LURIC study team who were either temporarily or permanently involved in patient recruitment as well as sample and data handling, in addition to the laboratory staff at the Ludwigshafen General Hospital and the Universities of Freiburg and Ulm, Germany. LURIC was supported by the 7th Framework Program RiskyCAD (grant agreement number 305739) of the European Union. The work of W.M. and M.E.K. is supported as part of the Competence Cluster of Nutrition and Cardiovascular Health (nutriCARD) which is funded by the German Federal Ministry of Education and Research.

**The Multi-Ethnic Study of Atherosclerosis (MESA)**

*Study Participants*

The Multi-Ethnic Study of Atherosclerosis (MESA) is a study of the characteristics of subclinical cardiovascular disease and the risk factors that predict progression to clinically overt cardiovascular disease or progression of the subclinical disease.[27] MESA consisted of a diverse, population-based sample of an initial 6,814 asymptomatic men and women aged 45-84. 38 percent of the recruited participants were white, 28 percent African American, 22 percent Hispanic, and 12 percent Asian, predominantly of Chinese descent. Participants were recruited from six field centers across the United States: Wake Forest University, Columbia University, Johns Hopkins University, University of Minnesota, Northwestern University and University of California - Los Angeles.

*MI and CHD Assessment*

Each participant received an extensive physical exam and assessment of subclinical measures of cardiovascular disease, standard coronary risk factors, sociodemographic factors, lifestyle factors, and psychosocial factors. Participants continue to be followed for identification and characterization of cardiovascular disease events, including acute myocardial infarction (MI) and other forms of coronary heart disease (CHD), stroke, and congestive heart failure; for cardiovascular disease interventions; and for mortality. The first examination took place over two years, from July 2000 - July 2002. It was followed by four examination periods that were 17-20 months in length. Participants have been contacted every 9 to 12 months throughout the study to assess clinical morbidity and mortality.

*Fibrinogen Measurement*

Fasting blood samples were collected, processed, and stored using standardized procedures. Fibrinogen antigen was measured using the BNII nephelometer (N Antiserum to Human Fibrinogen; Dade Behring Inc., Deerfield, IL). The assay was performed at the Laboratory for Clinical Biochemistry Research (University of Vermont, Burlington, VT). Intra- and inter-assay analytical coefficients of variation were 2.7% and 2.6%, respectively.

*Genotyping and imputation*

All participants were genotyped on the Affymetrix Genome-Wide Human SNP Array 6.0 (Affymetrix, Santa Clara, CA, USA) at the Affymetrix Research Services Lab. 6880 samples passed initial genotyping QC. African American samples were genotyped at the Broad Institute of Harvard and MIT as part of the CARe project. Affymetrix performed wet lab hybridization assay, and plate-based genotype calling using Birdseed v2. Sample QC was based on call rates and contrast QC (cQC) statistics. Broad performed similar QC for CARe sample. Additional sample and SNP QC were carried out at University of Virginia, including sample call rate, sample cQC, and sample heterozygosity by race at the sample level; Outlier plates checking by call rate, median cQC or heterozygosity at plate level. Four samples were removed due to low call rate (<95%). Cryptic sample duplicates or unresolved cryptic duplicates were dropped. Unresolved gender mismatches were also dropped. At the SNP level, we excluded monomorphic SNPs across all samples; SNPs with missing Rate was > 5% or observed heterozygosity > 53% were also excluded. Additional genotypes were imputed to the 1000 Genomes Phase I integrated variant set (NCBI build 37 / hg19) separately in each ethnic group using the program IMPUTE2. We used data freezes from 23 Nov 2010 (low-coverage whole-genome) and 21 May 2011 (high-coverage exome), phased haplotypes released March 2012 (v3), and phased haplotypes for 1,092 individuals and 39+ million variants. All imputed and genotyped SNPs were aligned to the '+' strand of the human genome reference sequence (NCBI Build 37).

*Acknowledgement*

MESA was supported by the Multi-Ethnic Study of Atherosclerosis (MESA) contracts N01-HC-95159, N01-HC-95160, N01-HC-95161, N01-HC-95162, N01-HC-95163, N01-HC-95164, N01-HC-95165, N01-HC-95166, N01-HC-95167, N01-HC-95168, N01-HC-95169 and by grants UL1-TR-000040 and UL1-RR-025005 from NCRR . Funding for MESA SHARe genotyping was provided by NHLBI Contract N02-HL-6-4278 and N01-HC65226. The provision of genotyping data was supported in part by the National Center for Advancing Translational Sciences, CTSI grant UL1TR001881, and the National Institute of Diabetes and Digestive and Kidney Disease Diabetes Research Center (DRC) grant DK063491 to the Southern California Diabetes Endocrinology Research Center.

**Prospective Study of Pravastatin in the Elderly at Risk** **(PROSPER)**

**The PROSPER study was only able to complete a portion of the analyses and thus does not appear in the main manuscript. However they contributed to initial analyses which helped set the direction of the overall study.**

*Study participants*

All data come from the PROspective Study of Pravastatin in the Elderly at Risk (PROSPER). A detailed description of the study has been published elsewhere. PROSPER was a prospective multicenter randomized placebo-controlled trial to assess whether treatment with pravastatin diminishes the risk of major vascular events in elderly. Between December 1997 and May 1999, we screened and enrolled subjects in Scotland (Glasgow), Ireland (Cork), and the Netherlands (Leiden). Men and women aged 70-82 years were recruited if they had pre-existing vascular disease or increased risk of such disease because of smoking, hypertension, or diabetes. A total number of 5,804 subjects were randomly assigned to pravastatin or placebo. A large number of prospective tests were performed including Biobank tests and cognitive function measurements.

*MI and CHD Assessment*

All endpoints were adjudicated by the PROSPER study endpoint committee. More details about the diagnosis of the coronary events within the PROSPER study has been published elsewhere.[28] Within this MR effort, MI is defined as fatal or non-fatal MI and CHD is defined as fatal and non-fatal MI, CHD death and revascularization procedures.

*Fibrinogen Measurement*

Fibrinogen levels were measured by the Clauss method[15] (MDA180 coagulometer; Trinity Biotech; calibrant 9th British standard National Institute for Biological Standards and Control).

*Genotyping and imputation*

A whole genome wide screening has been performed in the sequential PHASE project with the use of the Illumina 660K beadchip. Of 5,763 subjects DNA was available for genotyping. Genotyping was performed with the Illumina 660K beadchip, after QC (call rate <95%) 5,244 subjects and 557,192 SNPs were left for analysis. These SNPs were imputed to 2.5 million SNPs based on the HAPMAP build 36[29] with MACH[30] imputation software.

*Acknowledgments*

The research leading to these results has received funding from the European Union's Seventh Framework Programme (FP7/2007-2013) under grant agreement n° HEALTH-F2-2009-223004. For a part of the genotyping we received funding from the Netherlands Consortium of Healthy Aging (NGI: 05060810). This work was performed as part of an ongoing collaboration of the PROSPER study group in the universities of Leiden, Glasgow and Cork. Prof. Dr. J.W. Jukema is an Established Clinical Investigator of the Netherlands Heart Foundation (2001 D 032).

**Rotterdam Study (RS)**

*Study participants*

The Rotterdam Study is a prospective population-based cohort study initiated in 1990 to study the determinants of several chronic diseases in older adults.[31] The first cohort (RS-I) includes 7,983 inhabitants of Ommoord, a district of Rotterdam in the Netherlands, who were 55 years or older. The first examination took place between 1990 and 1993. The third examination, including 4,797 participants, took place between March 1997 and December 1999, and was used as the baseline in this study. Peripheral blood samples were taken at the third visit.

*MI and CHD Assessment*

CHD events were defined as fatal or non-fatal myocardial infarction, fatal CHD (possible and definite), and revascularization (percutaneous coronary artery intervention or coronary artery bypass grafting). The data collection process for cardiovascular outcomes has been described in detail previously.[32]

*Fibrinogen Measurement*

Fibrinogen levels were derived from the clotting curve of the prothrombin time assay using Thromborel S (Behringwerke, Marburg, Germany) as a reagent on an automated coagulation analyzer (Sysmex CA-500 Series Systems, Siemens, Breda, the Netherlands). Fibrinogen is reported in g/L.

*Genotyping and imputation*

Samples were genotyped using Infinium II HumanHap550 or 610 quad arrays. Before imputation, variants with a call rate less than 0.98, a minor allele frequency less than 1, or a Hardy-Weinberg equilibrium P-value less than 1×10^-6^ were excluded. Imputation was performed using the 1000 Genomes Project phase I version 3 reference panel with MaCH 1.0.15. Variants from the imputation were reported in variant dosage format ranging from 0-2.

**Study of Health in Pomerania (SHIP)**

*Study participants*

The Study of Health in Pomerania (SHIP) is a population-based project conducted in Greifswald, Germany.[33] Data from SHIP-0, SHIP-1, SHIP-2 were used. SHIP-0 was comprised of adult German residents in northeastern Germany living in three cities and 29 communities, with a total population of 212,157. A two-stage stratified cluster sample of adults aged 20-79 years at baseline was randomly drawn from local registries. The net sample (without migrated or deceased persons) comprised 6,267 eligible subjects, of which 4,308 Caucasian subjects participated at baseline (SHIP-0) between 1997 and 2001. The first follow-up was conducted 5 years later (SHIP-1; N=3300 subjects). From 2008 to 2012 the third phase of data collection (second follow-up examination, SHIP-2, N=2,333) was carried out.

*MI and CHD Assessment*

Information on non-fatal and fatal CV events was collected by self-report during follow-up interviews using a computer-assisted system, reports by participants’ general practitioners, information from electrocardiogram recordings and death certificates until end of October 2015 (the last day of data collected). For the current analysis, CHD was defined as the occurrence of an MI, angioplasty, coronary artery bypass graft, or CHD death.

*Fibrinogen Measurement*

Plasma fibrinogen concentrations were assayed according to Clauss (Electra analyzer, Instrumentation Laboratory, Barcelona, Spain). Fibrinogen is reported in g/L.

*Genotyping and imputation*

The SHIP samples were genotyped using the Affymetrix Genome-Wide Human SNP Array 6.0. Hybridisation of genomic DNA was done in accordance with the manufacturer’s standard recommendations. The genetic data analysis workflow was created using the Software InforSense. Genetic data were stored using the database Caché (InterSystems). Genotypes were determined using the Birdseed2 clustering algorithm. For quality control purposes, several control samples where added. On the chip level, only subjects with a genotyping rate on QC probesets (QC callrate) of at least 86% were included. Finally, all arrays had a sample callrate > 92%. The overall genotyping efficiency was 98.64 %. Imputation of genotypes in SHIP was performed with the software IMPUTE v2.2.2 based on the 1000Genomes Phase I (interim) panel released March 2012. Before imputation, SNPs with callreate <= 0.8 or HWE p-values < 1x10^-4^ were excluded.

*Analysis*

For regression analyses, SHIP data have been treated as interval-censored between the three SHIP examination dates SHIP0, SHIP1, SHIP2. If a study participant had no event (MI or CHD depending on the analysis), the interval was set to [SHIP2, Infinity). For study participants experiencing an event the interval was set to either (-Infinity, SHIP1] or [SHIP1, SHIP2], depending on when the event occurred. The computations have then been implemented with the *survreg* function for interval-censored data from the survival R package.

*Consent*

SHIP has been approved by the local ethics committee. After a complete description of the study to the subjects, written informed consent was obtained.

*Acknowledgments*

SHIP is part of the Community Medicine Research net of the University of Greifswald, Germany. This net is funded by the Federal Ministry of Education and Research (grants no. 01ZZ9603, 01ZZ0103, and 01ZZ0403), the Ministry of Cultural Affairs, as well as the Social Ministry of the Federal State of Mecklenburg-West Pomerania, and the network ‘Greifswald Approach to Individualized Medicine (GANI_MED)’ funded by the Federal Ministry of Education and Research (grant 03IS2061A). The University of Greifswald is a member of the ‘Center of Knowledge Interchange’ program of the Siemens AG and the Caché Campus program of the InterSystems GmbH.

**Women’s Genome Health Study (WGHS)**

*WGHS population*

The Women’s Genome Health Study (WGHS) is a prospective cohort of initially healthy, female North American health care professionals at least 45 years old at baseline representing participants in the Women’s Health Study (WHS) who provided a blood sample at baseline and consent for blood-based analyses.[34] The WHS was a 2x2 trial beginning in 1992-1994 of vitamin E and low dose aspirin in prevention of cancer and cardiovascular disease with about 10 years of follow-up. Since the end of the trial, follow-up has continued in observational mode. Additional information related to health and lifestyle were collected by questionnaire throughout the WHS trial and continuing observational follow-up.

*WGHS MI and CHD Assessment*

Assessment of MI and CHD in the WGHS was made via examinations of full medical records which are reviewed by a committee of physicians. WHO criteria were used to confirm myocardial infarction (MI) on the basis of abnormal cardiac enzymes or diagnostic electrocardiograms. Death certificates, autopsy reports, family reports, and medical records were used to determine cardiovascular deaths. All coronary revascularization procedures were confirmed by a medical record review.[34]

*Fibrinogen Assessment*

Fibrinogen was assessed via an immunoturbidimetric assay (Kamiya Biomedical, Seattle, Washington).[35] The assay was standardized using a calibrator provided by the World Health Organization.[36]

*WGHS genotyping*

Genotyping in the WGHS sample was performed using the HumanHap300 Duo ‘‘+’’ chips or the combination of the HumanHap300 Duo and iSelect chips (Illumina, San Diego, CA) with the Infinium II protocol. In either case, the custom SNP content was the same; these custom SNPs were chosen without regard to minor allele frequency (MAF) to saturate candidate genes for cardiovascular disease as well as to increase coverage of SNPs with known or suspected biological function, e.g. disease association, non-synonymous changes, substitutions at splice sites, etc. For quality control, all samples were required to have successful genotyping using the BeadStudio v. 3.3 software (Illumina, San Diego, CA) for at least 98% of the SNPs. A subset of 23,294 individuals were identified with self-reported European ancestry that could be verified on the basis of multidimensional scaling analysis of identity by state using1443 ancestry informative markers in PLINK v. 1.06. In the final dataset of these individuals, a total of 339596 SNPs were retained with MAF >1%, successful genotyping in 90% of the subjects, and deviations from Hardy-Weinberg equilibrium not exceeding P=10^-6^ in significance.

*1000 genomes imputation*

Among these same 23,294 individuals of verified European ancestry, genotypes for a total of 30,052,423 (autosomes) + 1,264,493 (X) SNPs were imputed from the experimental genotypes and phase information from the 1000G phase I v.3 release (March 2012) ALL panel using MaCH (v. 1.0.16) and Minimac (release 5/29/2012). A total of 332,927 genotyped SNPs that were selected by HWE p-value > 10^-6^ but unrestricted by MAF could be reconciled with the 1000G ALL panel and were used for imputation.

*Funding*

The WGHS is supported by the National Heart, Lung, and Blood Institute (HL043851 and HL080467) and the National Cancer Institute (CA047988 and UM1CA182913), the Donald W. Reynolds Foundation, and the Fondation Leducq, with collaborative scientific support and funding for genotyping provided by Amgen. Funding was also provided by ARRA grant HL099355.

**References**
